# Supplementary material for: Charge Transfer Chromophores Derived from 3d-Row Transition Metal Complexes
Source: Molecules. 2022 Nov 24;27(23):8175. doi: 10.3390/molecules27238175 (PMC9736222; doi:10.3390/molecules27238175)
Supplement: Supplementary file 1 [file molecules-27-08175-s001.zip › CatMDipy SI.pdf]

Charge transfer chromophores derived from 3d-row transition metal complexes

Kira I. Pashanova [1,*], Irina V. Ershova [1], Olesya Yu. Trofimova [1], Roman V. Rumyantsev [1], Georgy K. Fukin [1], Artem S. Bogomyakov [2], Maxim V. Arsenyev [1] and Alexandr V. Piskunov [1,*]

[1] Laboratory of Metal Complexes with Redox-Active Ligands, G.A. Razuvaev Institute of Organometallic Chemistry, Russian Academy of Sciences, 49 Tropinina Street, 603137 Nizhny Novgorod, Russia
[2] International Tomography Center, Siberian Branch of the Russian Academy of Sciences, 3a Institutskaya Street, 630090 Novosibirsk, Russia
* Correspondence: pashanova@iomc.ras.ru (K.I.P.); pial@iomc.ras.ru (A.V.P.); Tel.: +7-8312-462-77-09 (K.I.P. & A.V.P.)

Materials and methods

Initial reactants $Ni(cod)_2$, anhydrous $CoCl_2$, 4,4'-di-*tert*-butyl-2,2'-bipyridyl were commercial products (Sigma Aldrich), that is why used without additional purification. 3,6-di-*tert*-butyl-*o*-benzoquinone (**3,6-DTBQ**) was prepared in the course of current research in accordance with well-known synthetic technique – obtained results of elemental analysis and spectral parameters are in a good agreement with the exemplary data [1]. The solvents applied for synthetic and analytic manipulations were purified and dehydrated in accordance with conventional procedures [2, 3].

The elemental analysis was performed on Vario el Cube instrument. The IR-spectra were recorded on a FSM1201 Fourier-IR spectrometer in a Nujol in the range 4000–400 cm^{-1}. The ^1H NMR-spectrum for **2** was registered in $CDCl_3$ solution with a Bruker Avance Neo 300 MHz spectrometer, and the signals' positions were correlated with TMS as a standard. The EPR-spectrum for **1** was obtained applying a Bruker EMX spectrometer (9.75 GHz). The UV-vis-NIR spectra were registered in quartz cuvettes (path length l = 1 cm) on Shimadzu UV-3600 spectrophotometer within 200 – 2000 nm. Cyclic voltammetry experiments were implemented in three-electrode cell (Ag/AgCl/KCl reference electrode, platinum-flag auxiliary electrode, glassy carbon working electrode) with the 0.2 $V \cdot s^{-1}$ scanning rate in dried CH_2Cl_2, adding 0.2 M Bu_4NClO_4 buffer solution. The values of electrochemical potentials were referenced to the potential of Fc/Fc^+ redox pair.

DFT calculations for complexes 1 – 3

Density functional theory (DFT) calculations were performed using the Gaussian 09 program package [4] at the B3LYP/ 6-311++g(2d,2p) level. The stationary points on the potential energy surfaces were located by full geometry optimization with the calculation of the force constant matrix and checking for the stabilities of the DFT wave function.

Magnetic susceptibility for complex 3

The magnetic susceptibility measurements for the polycrystalline samples of complexes **1** – **3** were carried out with a Quantum Design MPMS*XL* SQUID magnetometer in the temperature range 2 – 300 K with magnetic field of up to 5 kOe. Diamagnetic corrections were made using the Pascal constants. The effective magnetic moment was calculated as $\mu_{eff}(T) = [(3k/N_A\mu_B^2)\chi T]^{1/2} \approx (8\chi T)^{1/2}$. Analysis of the experimental data was performed using PHI program [5].

Single-crystal X-ray diffraction studies for 1 – 3

The X-ray diffraction data for compounds **1-3** were collected on an Oxford Xcalibur Eos diffractometer (Mo-K_α radiation, ω-scan technique, λ = 0.71073 Å)*. The collected diffraction data was processed with the CrysAlisPro RED program [6]. All compounds were solved by dual method [7] and were refined on F^2_{hkl} using SHELXTL package [8]. All hydrogen atoms were placed in calculated positions. The hydrogen atoms of methyl-groups on special positions of complex **1·THF** were refined with DFIX restraints and $U_{iso}(H)$ = $1.5U_{eq}(C)$. All other hydrogen atoms in **1-3** were refined using a riding model ($U_{iso}(H) = 1.5U_{eq}(C)$ for CH_3-groups and $U_{iso}(H) = 1.2U_{eq}(C)$ for other groups). Two *tert*-butyl groups in each molecule of complexes **1-3** and all solvent molecules are disordered over two positions. Identical anisotropic displacement parameters for pairs of disordered atoms were received with EADP instruction. SADI, DFIX, FLAT, RIGU and ISOR instructions were additionally used to refine disordered fragments.

Table S1. X-ray diffraction data collection and structure refinement for complexes **1** – **3**.

Parameter	1·THF	2	3·toluene
Formula	$C_{36}H_{52}CuN_2O_3$	$C_{32}H_{44}N_2NiO_2$	$C_{71}H_{96}Co_2N_4O_4$
Formula weight	624.33	547.40	1187.37
Temperature (K)	298(2)	298(2)	298(2)
colour, habit	black, prism	black, plate	black, prism
Crystal size, mm	0.60 × 0.30 × 0.15	0.50 × 0.45 × 0.08	0.19 × 0.15 × 0.11
Crystal system	Orthorhombic	Monoclinic	Monoclinic
Space group	Pnma	$P2_1/c$	$P2_1/c$
Unit cell dimentions			
a, Å	23.4399(11)	28.9185(11)	13.6228(6)
b, Å	9.1189(5)	11.0826(3)	18.6411(6)
c, Å	16.7656(5)	20.3353(8)	14.4700(7)
α, deg*	90	90	90
β, deg	90	109.363(4)	113.538(6)
γ, deg*	90	90	90
V, $Å^3$	3583.6(3)	6148.7(4)	3368.8(3)
Z	4	8	2
density (calcd), g/cm^3	1.157	1.183	1.171
Absorption coefficient (mm^{-1})	0.643	0.660	0.541
F(000)	1340	2352	1272
θ range, deg	3.414 – 25.023	2.122 – 27.485	1.963 – 26.022
Index ranges	-19 ≤ h ≤ 27, -10 ≤ k ≤ 10, -19 ≤ l ≤ 19	-35 ≤ h ≤ 37, -14 ≤ k ≤ 13, -26 ≤ l ≤ 26	-16 ≤ h ≤ 16, -22 ≤ k ≤ 23, -17 ≤ l ≤ 17
Reflections collected	17731	61830	48857
Independent reflections	3372 [R_{int} = 0.0261]	14101 [R_{int} = 0.0361]	6624 [R_{int} = 0.0487]
Data/restraints/parameters	3372 / 166 / 323	14101 / 204 / 745	6624 / 401 / 405
R_1, wR_2 [I>2σ(I)]	0.0439, 0.1177	0.0439, 0.0971	0.0489, 0.1173
R_1, wR_2 (all data)	0.0596, 0.1287	0.0726, 0.1099	0.0710, 0.1291
goodness-of-fit on F^2	1.021	1.010	1.052
T_{min} / T_{max}*	0.84928 / 0.85025	0.84510 / 0.84647	0.936 / 0.936
Extinction coefficient			
$\Delta\rho_{max}/\Delta\rho_{min}$ (e/$Å^3$)	0.429 / -0.354	0.347 / -0.262	0.412 / -0.334

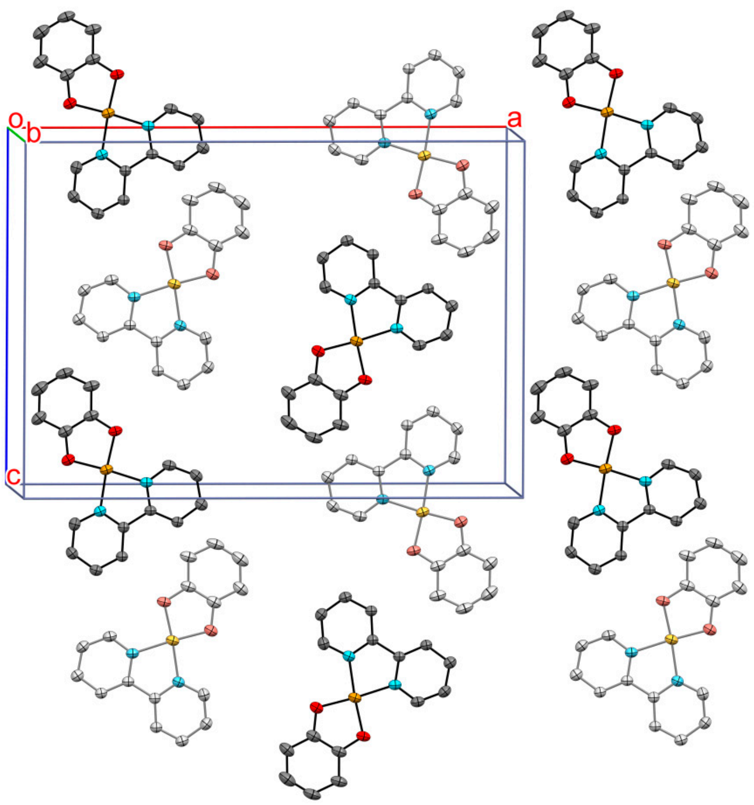

Figure S1. The fragment of crystal packing for complex **1** – the molecules from odd and even layers are represented as bright and faded pictures, respectively. Structures are given with 30% thermal probability ellipsoids. Hydrogen atoms, *tert*-butyl groups and THF molecules are omitted for clarity.

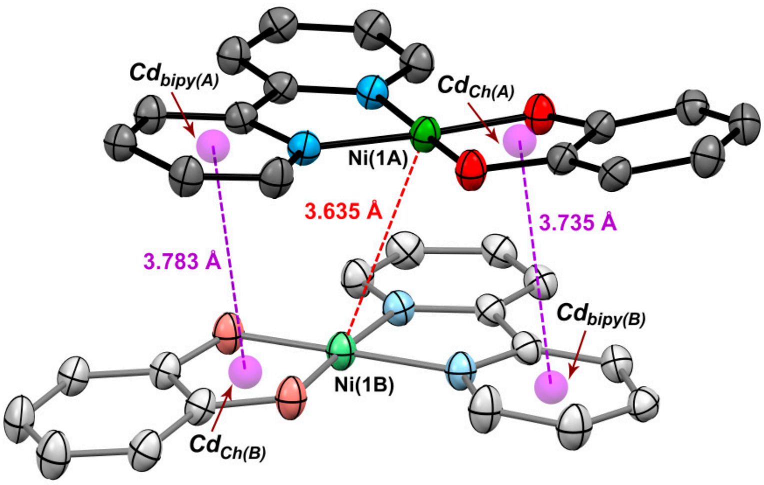

Figure S2. The shortest intra-stack contacts $Cd_{\mathrm{Cat(A)}}\cdots Cd_{\mathrm{bipy(B)}}$, $Cd_{\mathrm{Cat(B)}}\cdots Cd_{\mathrm{bipy(A)}}$, and Ni(1A)···Ni(1B) for complex **2**. Structures are given with 30% thermal probability ellipsoids. Hydrogen atoms and *tert*-butyl groups are omitted for clarity.

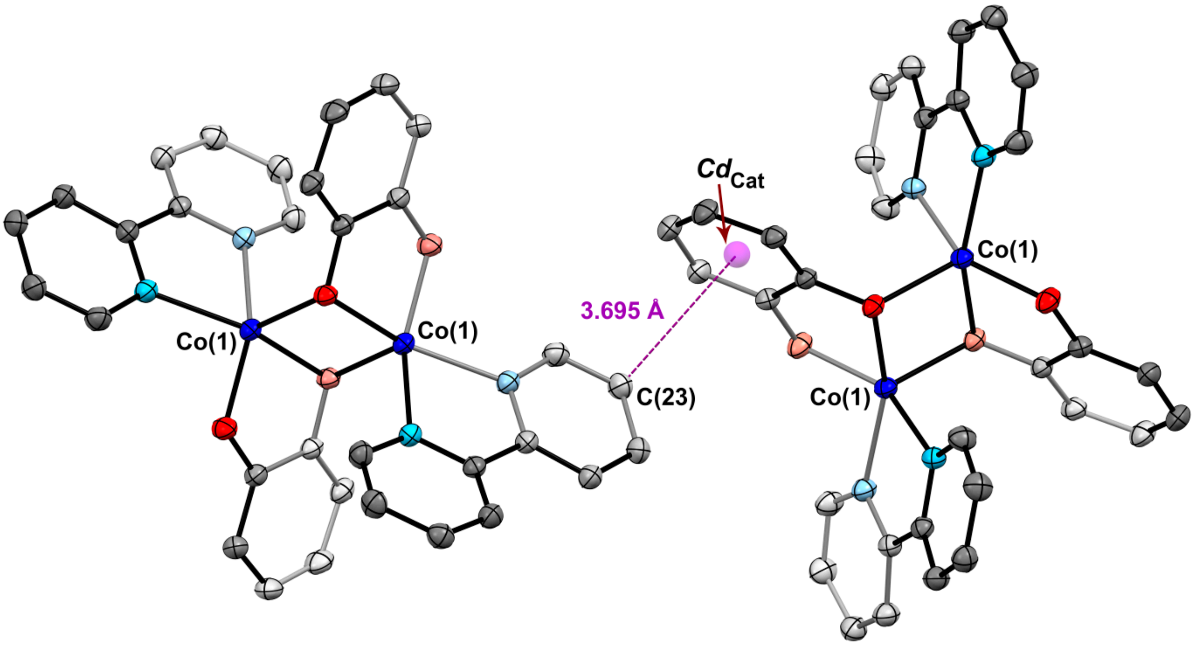

Figure S3. The shortest contact $Cd_{\mathrm{Cat}}\cdots$C(23) = 3.695 Å for complex **3**. Structures are given with 30% thermal probability ellipsoids. Hydrogen atoms and *tert*-butyl groups are omitted for clarity.

References

1. Fukin, G., et al., *Geometrical and energetical aspects of structure of 3, 6-di-tert-butyl-o-benzoquinones.* Structural Chemistry, 2010. **21**(3): p. 607-611.

2. Reichardt, C. and T. Welton, *Solvents and solvent effects in organic chemistry*, ed. C. Reichardt and T. Welton. 2011: John Wiley & Sons.

3. Gordon, A. and R. Ford, *The Chemist's Companion, Wiley.* New York, 1972: p. 143.

4. M. J. Frisch, G. W. Trucks, H. B. Schlegel, G. E. Scuseria, M. A. Robb, J. Cheeseman, R. Scalmani, V. Barone, B. Mennucci G.A. Petersson, H. Nakatsuji, M. Caricato, X. Li, H. P. Hratchian, A. F. Izmaylov, J. Bloino, G. Zheng, J. L. Sonnenberg, M. Hada, M. Ehara, K. Toyota, R. Fukuda, J. Hasegawa, M. Ishida, T. Nakajima, Y. Honda, O. Kitao, H. Nakai, T. Vreven, J.A.M. Jr., J.E. Peralta, F. Ogliaro, M. Bearpark, J. J. Heyd, E. Brothers, K. N. Kudin, V. N. Staroverov, T. Keith, R. Kobayashi, J. Normand, K. Raghavachari, A. Rendell, J.C. Burant, S.S. Iyengar, J. Tomasi, M. Cossi, N. Rega, J.M. Millam, M. Klene, J.E. Knox, J.B. Cross, V. Bakken, C. Adamo, J. Jaramillo, R. Gomperts, R.E. Stratmann, O. Yazyev, A.J. Austin, R. Cammi, C. Pomelli, J.W. Ochterski, R.L. Martin, K. Morokuma, V.G. Zakrzewski, G.A. Voth, P. Salvador, J.J. Dannenberg, S. Dapprich, A.D. Daniels, O. Farkas, J.B. Foresman, J.V. Ortiz, J. Cioslowski, D.J.Fox, GAUSSIAN 09. Revision D.01, Gaussian, Inc., Wallingford CT, – 2013.

5. Chilton, N.F., et al., *PHI: A powerful new program for the analysis of anisotropic monomeric and exchange-coupled polynuclear d-and f-block complexes.* Journal of Computational Chemistry, 2013. **34**(13): p. 1164-1175.

6. Rigaku Oxford Diffraction. (2021). CrysAlis Pro software system, version 1.171.41.122a, Rigaku Corporation, Wroclaw, Poland.

7. G. M. Sheldrick, Acta Crystallogr., Sect. A: Found. Adv., 2015, 71, 3.

8. G. M. Sheldrick, Acta Crystallogr., Sect. C: Struct. Chem., 2015, 71, 3.
