# Supplementary material for: Charge Transfer Chromophores Derived from 3d-Row Transition Metal Complexes
Source: Molecules. 2022 Nov 24;27(23):8175. doi: 10.3390/molecules27238175 (PMC9736222; doi:10.3390/molecules27238175)

checkCIF/PLATON report

You have not supplied any structure factors. As a result the full set of tests cannot be run.

THIS REPORT IS FOR GUIDANCE ONLY. IF USED AS PART OF A REVIEW PROCEDURE FOR PUBLICATION, IT SHOULD NOT REPLACE THE EXPERTISE OF AN EXPERIENCED CRYSTALLOGRAPHIC REFEREE.

No syntax errors found. CIF dictionary Interpreting this report

Datablock: cucatbipy

```
Bond precision:   C-C = 0.0057 A          Wavelength=0.71073

Cell:              a=23.4399(11)      b=9.1189(5)      c=16.7656(5)
                   alpha=90           beta=90          gamma=90
Temperature:       298 K

                Calculated                    Reported
Volume          3583.6(3)                     3583.6(3)
Space group     P n m a                       P n m a
Hall group      -P 2ac 2n                     -P 2ac 2n
Moiety formula  C32 H44 Cu N2 O2, C4 H8 O    ?
Sum formula     C36 H52 Cu N2 O3              C36 H52 Cu N2 O3
Mr              624.35                        624.33
Dx,g cm-3       1.157                         1.157
Z               4                             4
Mu (mm-1)       0.643                         0.643
F000            1340.0                        1340.0
F000'           1341.71
h,k,lmax        27,10,19                      27,10,19
Nref            3383                          3372
Tmin,Tmax                                     0.849,0.850
Tmin'

Correction method= # Reported T Limits: Tmin=0.849 Tmax=0.850
AbsCorr = SPHERE

Data completeness= 0.997           Theta(max)= 25.023

R(reflections)= 0.0439( 2609)                    wR2(reflections)=
                                                 0.1287( 3372)
S = 1.021                  Npar= 323
```

The following ALERTS were generated. Each ALERT has the format
test-name_ALERT_alert-type_alert-level.
Click on the hyperlinks for more details of the test.

Alert level C

```
PLAT220_ALERT_2_C NonSolvent   Resd 1  C   Ueq(max)/Ueq(min) Range        3.5 Ratio
PLAT242_ALERT_2_C Low    ’MainMol’ Ueq as Compared to Neighbors of        C7 Check
PLAT242_ALERT_2_C Low    ’MainMol’ Ueq as Compared to Neighbors of       C10 Check
PLAT242_ALERT_2_C Low    ’MainMol’ Ueq as Compared to Neighbors of       C23 Check
PLAT242_ALERT_2_C Low    ’MainMol’ Ueq as Compared to Neighbors of       C26 Check
```

Alert level G

```
PLAT002_ALERT_2_G Number of Distance or Angle Restraints on AtSite         36 Note
PLAT003_ALERT_2_G Number of Uiso or Uij Restrained non-H Atoms ...         18 Report
PLAT164_ALERT_4_G Nr. of Refined C-H H-Atoms in Heavy-Atom Struct.          4 Note
PLAT171_ALERT_4_G The CIF-Embedded .res File Contains EADP Records          9 Report
PLAT172_ALERT_4_G The CIF-Embedded .res File Contains DFIX Records         30 Report
PLAT186_ALERT_4_G The CIF-Embedded .res File Contains ISOR Records          3 Report
PLAT230_ALERT_2_G Hirshfeld Test Diff for    C23      --C24      .       11.7 s.u.
PLAT230_ALERT_2_G Hirshfeld Test Diff for    C23      --C24’     .        9.0 s.u.
PLAT230_ALERT_2_G Hirshfeld Test Diff for    C23      --C25’     .       11.7 s.u.
PLAT230_ALERT_2_G Hirshfeld Test Diff for    C26      --C27      .        8.8 s.u.
PLAT230_ALERT_2_G Hirshfeld Test Diff for    C26      --C27’     .       11.7 s.u.
PLAT230_ALERT_2_G Hirshfeld Test Diff for    C26      --C28’     .        5.7 s.u.
PLAT301_ALERT_3_G Main Residue  Disorder .............(Resd  1  )        21% Note
PLAT302_ALERT_4_G Anion/Solvent/Minor-Residue Disorder (Resd  2  )       100% Note
PLAT302_ALERT_4_G Anion/Solvent/Minor-Residue Disorder (Resd  3  )       100% Note
PLAT304_ALERT_4_G Non-Integer Number of Atoms in ..... (Resd  2  )       2.13 Check
PLAT304_ALERT_4_G Non-Integer Number of Atoms in ..... (Resd  3  )       4.37 Check
PLAT367_ALERT_2_G Long?  C(sp?)-C(sp?) Bond  C15      - C23      .       1.52 Ang.
PLAT367_ALERT_2_G Long?  C(sp?)-C(sp?) Bond  C20      - C26      .       1.53 Ang.
PLAT398_ALERT_2_G Deviating  C-O-C   Angle From 120 for O1S      .      107.2 Degree
PLAT410_ALERT_2_G Short Intra H...H Contact  H16A     ..H24C     .       1.64 Ang.
                                                      x,y,z  =      1_555 Check
PLAT410_ALERT_2_G Short Intra H...H Contact  H19A     ..H27C     .       1.71 Ang.
                                                      x,y,z  =      1_555 Check
PLAT720_ALERT_4_G Number of Unusual/Non-Standard Labels ..........         16 Note
PLAT721_ALERT_1_G Bond    Calc     0.96000, Rep     0.97000 Dev...       0.01 Ang.
              C3Q      -H3QA            1_555   1_555 ........     #  108 Check
PLAT789_ALERT_4_G Atoms with Negative _atom_site_disorder_group  #         26 Check
PLAT860_ALERT_3_G Number of Least-Squares Restraints ............         166 Note
PLAT965_ALERT_2_G The SHELXL WEIGHT Optimisation has not Converged     Please Check
```

0 **ALERT level A** = Most likely a serious problem - resolve or explain
0 **ALERT level B** = A potentially serious problem, consider carefully
5 **ALERT level C** = Check. Ensure it is not caused by an omission or oversight
27 **ALERT level G** = General information/check it is not something unexpected

1 ALERT type 1 CIF construction/syntax error, inconsistent or missing data
19 ALERT type 2 Indicator that the structure model may be wrong or deficient
2 ALERT type 3 Indicator that the structure quality may be low
10 ALERT type 4 Improvement, methodology, query or suggestion
0 ALERT type 5 Informative message, check

It is advisable to attempt to resolve as many as possible of the alerts in all categories. Often the minor alerts point to easily fixed oversights, errors and omissions in your CIF or refinement strategy, so attention to these fine details can be worthwhile. In order to resolve some of the more serious problems it may be necessary to carry out additional measurements or structure refinements. However, the purpose of your study may justify the reported deviations and the more serious of these should normally be commented upon in the discussion or experimental section of a paper or in the "special_details" fields of the CIF. checkCIF was carefully designed to identify outliers and unusual parameters, but every test has its limitations and alerts that are not important in a particular case may appear. Conversely, the absence of alerts does not guarantee there are no aspects of the results needing attention. It is up to the individual to critically assess their own results and, if necessary, seek expert advice.

Publication of your CIF in IUCr journals

A basic structural check has been run on your CIF. These basic checks will be run on all CIFs submitted for publication in IUCr journals (*Acta Crystallographica*, *Journal of Applied Crystallography*, *Journal of Synchrotron Radiation*); however, if you intend to submit to *Acta Crystallographica Section C* or *E* or *IUCrData*, you should make sure that full publication checks are run on the final version of your CIF prior to submission.

Publication of your CIF in other journals

Please refer to the *Notes for Authors* of the relevant journal for any special instructions relating to CIF submission.

PLATON version of 12/09/2022; check.def file version of 09/08/2022

Datablock cucatbipy - ellipsoid plot

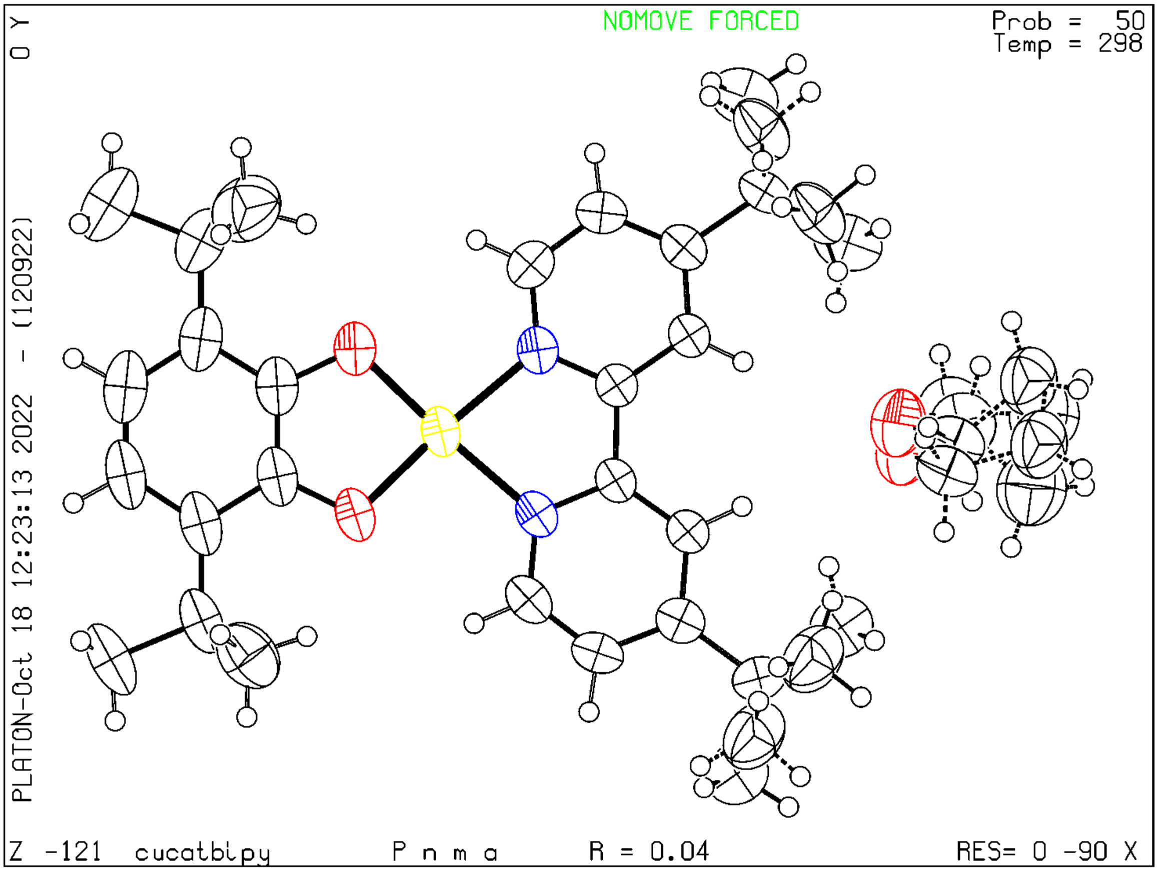

Supplement: Supplementary file 1 [file molecules-27-08175-s001.zip › Complex 1.pdf]
