# Supplementary material for: Charge Transfer Chromophores Derived from 3d-Row Transition Metal Complexes
Source: Molecules. 2022 Nov 24;27(23):8175. doi: 10.3390/molecules27238175 (PMC9736222; doi:10.3390/molecules27238175)

checkCIF/PLATON report

You have not supplied any structure factors. As a result the full set of tests cannot be run.

THIS REPORT IS FOR GUIDANCE ONLY. IF USED AS PART OF A REVIEW PROCEDURE FOR PUBLICATION, IT SHOULD NOT REPLACE THE EXPERTISE OF AN EXPERIENCED CRYSTALLOGRAPHIC REFEREE.

No syntax errors found. CIF dictionary Interpreting this report

Datablock: nicatbipytbu

```
Bond precision:   C-C = 0.0034 A          Wavelength=0.71073

Cell:             a=28.9185(11)     b=11.0826(3)       c=20.3353(8)
                  alpha=90          beta=109.363(4)    gamma=90
Temperature:      298 K

                  Calculated                       Reported
Volume            6148.7(4)                        6148.7(4)
Space group       P 21/c                           P 21/c
Hall group        -P 2ybc                          -P 2ybc
Moiety formula    C32 H44 N2 Ni O2                 ?
Sum formula       C32 H44 N2 Ni O2                 C32 H44 N2 Ni O2
Mr                547.38                           547.40
Dx,g cm-3         1.183                            1.183
Z                 8                                8
Mu (mm-1)         0.660                            0.660
F000              2352.0                           2352.0
F000'             2355.43
h,k,lmax          37,14,26                         37,14,26
Nref              14111                            14101
Tmin,Tmax                                          0.845,0.846
Tmin'

Correction method= # Reported T Limits: Tmin=0.845 Tmax=0.846
AbsCorr = SPHERE

Data completeness= 0.999            Theta(max)= 27.485

                                                       wR2(reflections)=
R(reflections)= 0.0439( 10064)                         0.1099( 14101)

S = 1.010                     Npar= 745
```

The following ALERTS were generated. Each ALERT has the format
test-name_ALERT_alert-type_alert-level.
Click on the hyperlinks for more details of the test.

Alert level C

```
PLAT242_ALERT_2_C Low    'MainMol' Ueq as Compared to Neighbors of        C7A Check
PLAT242_ALERT_2_C Low    'MainMol' Ueq as Compared to Neighbors of       C11A Check
PLAT242_ALERT_2_C Low    'MainMol' Ueq as Compared to Neighbors of       C29A Check
PLAT242_ALERT_2_C Low    'MainMol' Ueq as Compared to Neighbors of        C7B Check
PLAT242_ALERT_2_C Low    'MainMol' Ueq as Compared to Neighbors of       C25B Check
PLAT242_ALERT_2_C Low    'MainMol' Ueq as Compared to Neighbors of       C29B Check
PLAT601_ALERT_2_C Unit Cell Contains Solvent Accessible VOIDS of .         38 Ang**3
```

Alert level G

```
PLAT002_ALERT_2_G Number of Distance or Angle Restraints on AtSite         28 Note
PLAT003_ALERT_2_G Number of Uiso or Uij Restrained non-H Atoms ...         26 Report
PLAT171_ALERT_4_G The CIF-Embedded .res File Contains EADP Records         12 Report
PLAT172_ALERT_4_G The CIF-Embedded .res File Contains DFIX Records         16 Report
PLAT186_ALERT_4_G The CIF-Embedded .res File Contains ISOR Records          5 Report
PLAT230_ALERT_2_G Hirshfeld Test Diff for    C29A     --C30'     .       10.3 s.u.
PLAT230_ALERT_2_G Hirshfeld Test Diff for    C29A     --C32'     .        6.0 s.u.
PLAT230_ALERT_2_G Hirshfeld Test Diff for    C25B     --C26"     .        6.0 s.u.
PLAT230_ALERT_2_G Hirshfeld Test Diff for    C25B     --C27"     .        6.0 s.u.
PLAT230_ALERT_2_G Hirshfeld Test Diff for    C25B     --C28"     .       11.7 s.u.
PLAT230_ALERT_2_G Hirshfeld Test Diff for    C29B     --C30"     .       17.3 s.u.
PLAT230_ALERT_2_G Hirshfeld Test Diff for    C29B     --C31"     .        8.3 s.u.
PLAT230_ALERT_2_G Hirshfeld Test Diff for    C29B     --C32"     .       10.3 s.u.
PLAT301_ALERT_3_G Main Residue  Disorder ..............(Resd  1  )        16% Note
PLAT301_ALERT_3_G Main Residue  Disorder ..............(Resd  2  )        16% Note
PLAT412_ALERT_2_G Short Intra XH3 .. XHn     H18A     ..H27A     .       2.08 Ang.
                                                      x,y,z  =      1_555 Check
PLAT412_ALERT_2_G Short Intra XH3 .. XHn     H23A     ..H30F     .       1.95 Ang.
                                                      x,y,z  =      1_555 Check
PLAT412_ALERT_2_G Short Intra XH3 .. XHn     H21B     ..H32H     .       2.13 Ang.
                                                      x,y,z  =      1_555 Check
PLAT412_ALERT_2_G Short Intra XH3 .. XHn     H23B     ..H30I     .       2.13 Ang.
                                                      x,y,z  =      1_555 Check
PLAT720_ALERT_4_G Number of Unusual/Non-Standard Labels ..........         16 Note
PLAT794_ALERT_5_G Tentative Bond Valency for Ni1A      (III)     .       3.04 Info
PLAT794_ALERT_5_G Tentative Bond Valency for Ni1B      (III)     .       3.04 Info
PLAT860_ALERT_3_G Number of Least-Squares Restraints .............        204 Note
PLAT933_ALERT_2_G Number of HKL-OMIT Records in Embedded .res File          1 Note
PLAT941_ALERT_3_G Average HKL Measurement Multiplicity ...........        4.4 Low
PLAT965_ALERT_2_G The SHELXL WEIGHT Optimisation has not Converged     Please Check
```

---

0 **ALERT level A** = Most likely a serious problem - resolve or explain
0 **ALERT level B** = A potentially serious problem, consider carefully
7 **ALERT level C** = Check. Ensure it is not caused by an omission or oversight
26 **ALERT level G** = General information/check it is not something unexpected

0 ALERT type 1 CIF construction/syntax error, inconsistent or missing data

```
 23 ALERT type 2 Indicator that the structure model may be wrong or deficient
  4 ALERT type 3 Indicator that the structure quality may be low
  4 ALERT type 4 Improvement, methodology, query or suggestion
  2 ALERT type 5 Informative message, check
```

It is advisable to attempt to resolve as many as possible of the alerts in all categories. Often the minor alerts point to easily fixed oversights, errors and omissions in your CIF or refinement strategy, so attention to these fine details can be worthwhile. In order to resolve some of the more serious problems it may be necessary to carry out additional measurements or structure refinements. However, the purpose of your study may justify the reported deviations and the more serious of these should normally be commented upon in the discussion or experimental section of a paper or in the "special_details" fields of the CIF. checkCIF was carefully designed to identify outliers and unusual parameters, but every test has its limitations and alerts that are not important in a particular case may appear. Conversely, the absence of alerts does not guarantee there are no aspects of the results needing attention. It is up to the individual to critically assess their own results and, if necessary, seek expert advice.

Publication of your CIF in IUCr journals

A basic structural check has been run on your CIF. These basic checks will be run on all CIFs submitted for publication in IUCr journals (*Acta Crystallographica*, *Journal of Applied Crystallography*, *Journal of Synchrotron Radiation*); however, if you intend to submit to *Acta Crystallographica Section C* or *E* or *IUCrData*, you should make sure that full publication checks are run on the final version of your CIF prior to submission.

Publication of your CIF in other journals

Please refer to the *Notes for Authors* of the relevant journal for any special instructions relating to CIF submission.

PLATON version of 12/09/2022; check.def file version of 09/08/2022

Datablock nicatbipytbu - ellipsoid plot

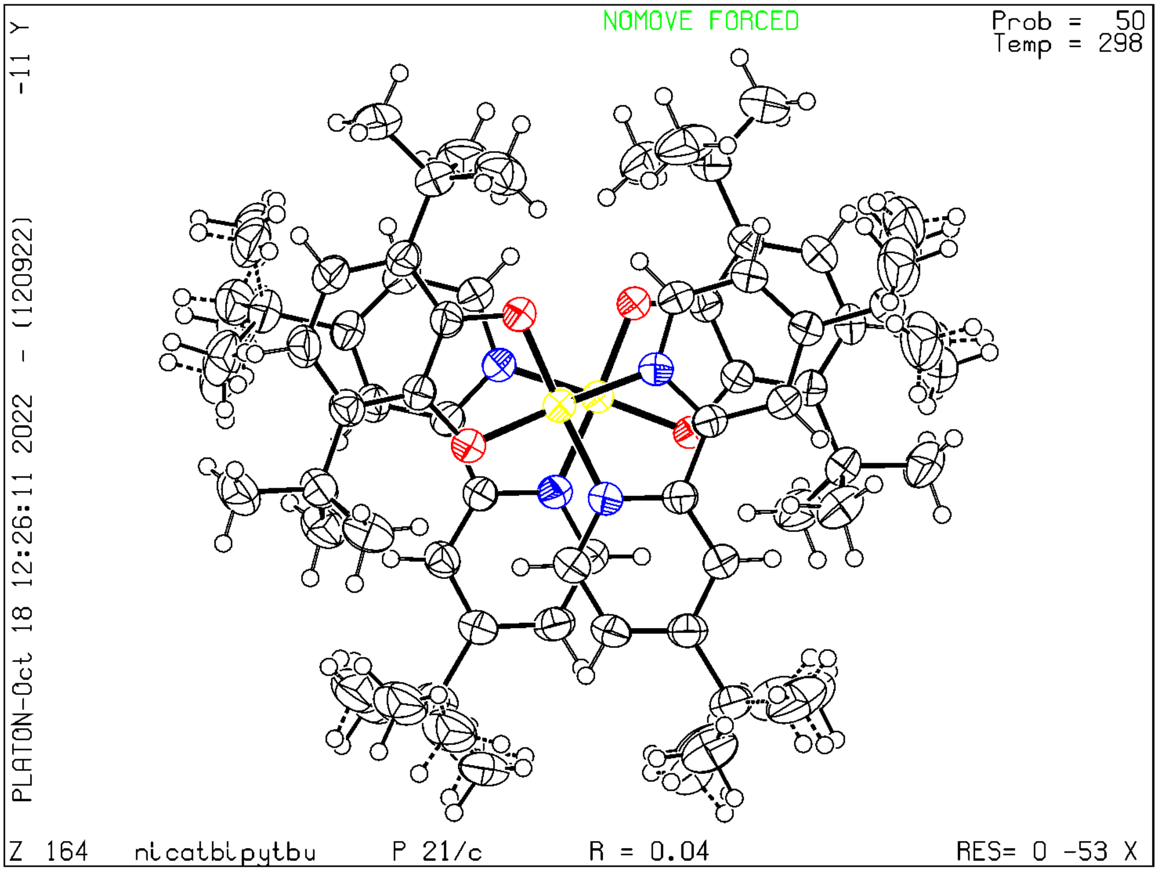

Supplement: Supplementary file 1 [file molecules-27-08175-s001.zip › complex 2.pdf]
