# Supplementary material for: Charge Transfer Chromophores Derived from 3d-Row Transition Metal Complexes
Source: Molecules. 2022 Nov 24;27(23):8175. doi: 10.3390/molecules27238175 (PMC9736222; doi:10.3390/molecules27238175)

checkCIF/PLATON report

You have not supplied any structure factors. As a result the full set of tests cannot be run.

THIS REPORT IS FOR GUIDANCE ONLY. IF USED AS PART OF A REVIEW PROCEDURE FOR PUBLICATION, IT SHOULD NOT REPLACE THE EXPERTISE OF AN EXPERIENCED CRYSTALLOGRAPHIC REFEREE.

No syntax errors found. CIF dictionary Interpreting this report

Datablock: cosqbpytbu

```
Bond precision:   C-C = 0.0048 A          Wavelength=0.71073

Cell:             a=13.6228(6)     b=18.6411(6)        c=14.4700(7)
                  alpha=90         beta=113.538(6)     gamma=90
Temperature:      298 K

                 Calculated                          Reported
Volume           3368.8(3)                           3368.8(3)
Space group      P 21/c                              P 21/c
Hall group       -P 2ybc                             -P 2ybc
Moiety formula   C64 H88 Co2 N4 O4, C7 H8            C64 H88 Co2 N4 O4, C7 H8
Sum formula      C71 H96 Co2 N4 O4                   C71 H96 Co2 N4 O4
Mr               1187.38                             1187.37
Dx,g cm-3        1.171                               1.171
Z                2                                   2
Mu (mm-1)        0.541                               0.541
F000             1272.0                              1272.0
F000'            1273.79
h,k,lmax         16,23,17                            16,23,17
Nref             6629                                6624
Tmin,Tmax        0.907,0.942                         0.936,0.936
Tmin'            0.902

Correction method= # Reported T Limits: Tmin=0.936 Tmax=0.936
AbsCorr = MULTI-SCAN

Data completeness= 0.999           Theta(max)= 26.022

                                                   wR2(reflections)=
R(reflections)= 0.0489( 5079)                      0.1291( 6624)

S = 1.052                  Npar= 405
```

The following ALERTS were generated. Each ALERT has the format
test-name_ALERT_alert-type_alert-level.
Click on the hyperlinks for more details of the test.

Alert level C

```
PLAT220_ALERT_2_C NonSolvent   Resd 1  C   Ueq(max)/Ueq(min) Range        4.1 Ratio
PLAT222_ALERT_3_C NonSolvent Resd 1  H   Uiso(max)/Uiso(min) Range        4.1 Ratio
PLAT242_ALERT_2_C Low    'MainMol' Ueq as Compared to Neighbors of        C11 Check
PLAT242_ALERT_2_C Low    'MainMol' Ueq as Compared to Neighbors of        C25 Check
PLAT242_ALERT_2_C Low    'MainMol' Ueq as Compared to Neighbors of        C29 Check
PLAT260_ALERT_2_C Large Average Ueq of Residue Including       C1S      0.142 Check
```

Alert level G

```
PLAT002_ALERT_2_G Number of Distance or Angle Restraints on AtSite         11 Note
PLAT003_ALERT_2_G Number of Uiso or Uij Restrained non-H Atoms ...          9 Report
PLAT172_ALERT_4_G The CIF-Embedded .res File Contains DFIX Records          5 Report
PLAT174_ALERT_4_G The CIF-Embedded .res File Contains FLAT Records          1 Report
PLAT176_ALERT_4_G The CIF-Embedded .res File Contains SADI Records          1 Report
PLAT186_ALERT_4_G The CIF-Embedded .res File Contains ISOR Records          3 Report
PLAT187_ALERT_4_G The CIF-Embedded .res File Contains RIGU Records          1 Report
PLAT300_ALERT_4_G Atom Site Occupancy of C1S       Constrained at         0.5 Check
PLAT300_ALERT_4_G Atom Site Occupancy of C2S       Constrained at         0.5 Check
PLAT300_ALERT_4_G Atom Site Occupancy of C3S       Constrained at         0.5 Check
PLAT300_ALERT_4_G Atom Site Occupancy of C4S       Constrained at         0.5 Check
PLAT300_ALERT_4_G Atom Site Occupancy of C5S       Constrained at         0.5 Check
PLAT300_ALERT_4_G Atom Site Occupancy of C6S       Constrained at         0.5 Check
PLAT300_ALERT_4_G Atom Site Occupancy of C7S       Constrained at         0.5 Check
PLAT300_ALERT_4_G Atom Site Occupancy of H2SA      Constrained at         0.5 Check
PLAT300_ALERT_4_G Atom Site Occupancy of H3SA      Constrained at         0.5 Check
PLAT300_ALERT_4_G Atom Site Occupancy of H4SA      Constrained at         0.5 Check
PLAT300_ALERT_4_G Atom Site Occupancy of H5SA      Constrained at         0.5 Check
PLAT300_ALERT_4_G Atom Site Occupancy of H6SA      Constrained at         0.5 Check
PLAT300_ALERT_4_G Atom Site Occupancy of H7SA      Constrained at         0.5 Check
PLAT300_ALERT_4_G Atom Site Occupancy of H7SB      Constrained at         0.5 Check
PLAT300_ALERT_4_G Atom Site Occupancy of H7SC      Constrained at         0.5 Check
PLAT301_ALERT_3_G Main Residue  Disorder .............(Resd  1  )          8% Note
PLAT302_ALERT_4_G Anion/Solvent/Minor-Residue Disorder (Resd  2  )        100% Note
PLAT304_ALERT_4_G Non-Integer Number of Atoms in ..... (Resd  2  )       7.50 Check
PLAT380_ALERT_4_G Incorrectly? Oriented X(sp2)-Methyl Moiety .....        C7S Check
PLAT412_ALERT_2_G Short Intra XH3 .. XHn     H16A     ..H26D     .       2.13 Ang.
                                                       x,y,z  =      1_555 Check
PLAT720_ALERT_4_G Number of Unusual/Non-Standard Labels ..........         11 Note
PLAT789_ALERT_4_G Atoms with Negative _atom_site_disorder_group  #         15 Check
PLAT794_ALERT_5_G Tentative Bond Valency for Co1       (II)      .       1.99 Info
PLAT860_ALERT_3_G Number of Least-Squares Restraints .............        401 Note
PLAT965_ALERT_2_G The SHELXL WEIGHT Optimisation has not Converged     Please Check
```

0 **ALERT level A** = Most likely a serious problem - resolve or explain
0 **ALERT level B** = A potentially serious problem, consider carefully
6 **ALERT level C** = Check. Ensure it is not caused by an omission or oversight
32 **ALERT level G** = General information/check it is not something unexpected

0 ALERT type 1 CIF construction/syntax error, inconsistent or missing data

```
 9 ALERT type 2 Indicator that the structure model may be wrong or deficient
 3 ALERT type 3 Indicator that the structure quality may be low
25 ALERT type 4 Improvement, methodology, query or suggestion
 1 ALERT type 5 Informative message, check
```

It is advisable to attempt to resolve as many as possible of the alerts in all categories. Often the minor alerts point to easily fixed oversights, errors and omissions in your CIF or refinement strategy, so attention to these fine details can be worthwhile. In order to resolve some of the more serious problems it may be necessary to carry out additional measurements or structure refinements. However, the purpose of your study may justify the reported deviations and the more serious of these should normally be commented upon in the discussion or experimental section of a paper or in the "special_details" fields of the CIF. checkCIF was carefully designed to identify outliers and unusual parameters, but every test has its limitations and alerts that are not important in a particular case may appear. Conversely, the absence of alerts does not guarantee there are no aspects of the results needing attention. It is up to the individual to critically assess their own results and, if necessary, seek expert advice.

Publication of your CIF in IUCr journals

A basic structural check has been run on your CIF. These basic checks will be run on all CIFs submitted for publication in IUCr journals (*Acta Crystallographica*, *Journal of Applied Crystallography*, *Journal of Synchrotron Radiation*); however, if you intend to submit to *Acta Crystallographica Section C* or *E* or *IUCrData*, you should make sure that full publication checks are run on the final version of your CIF prior to submission.

Publication of your CIF in other journals

Please refer to the *Notes for Authors* of the relevant journal for any special instructions relating to CIF submission.

PLATON version of 12/09/2022; check.def file version of 09/08/2022

Datablock cosqbpytbu - ellipsoid plot

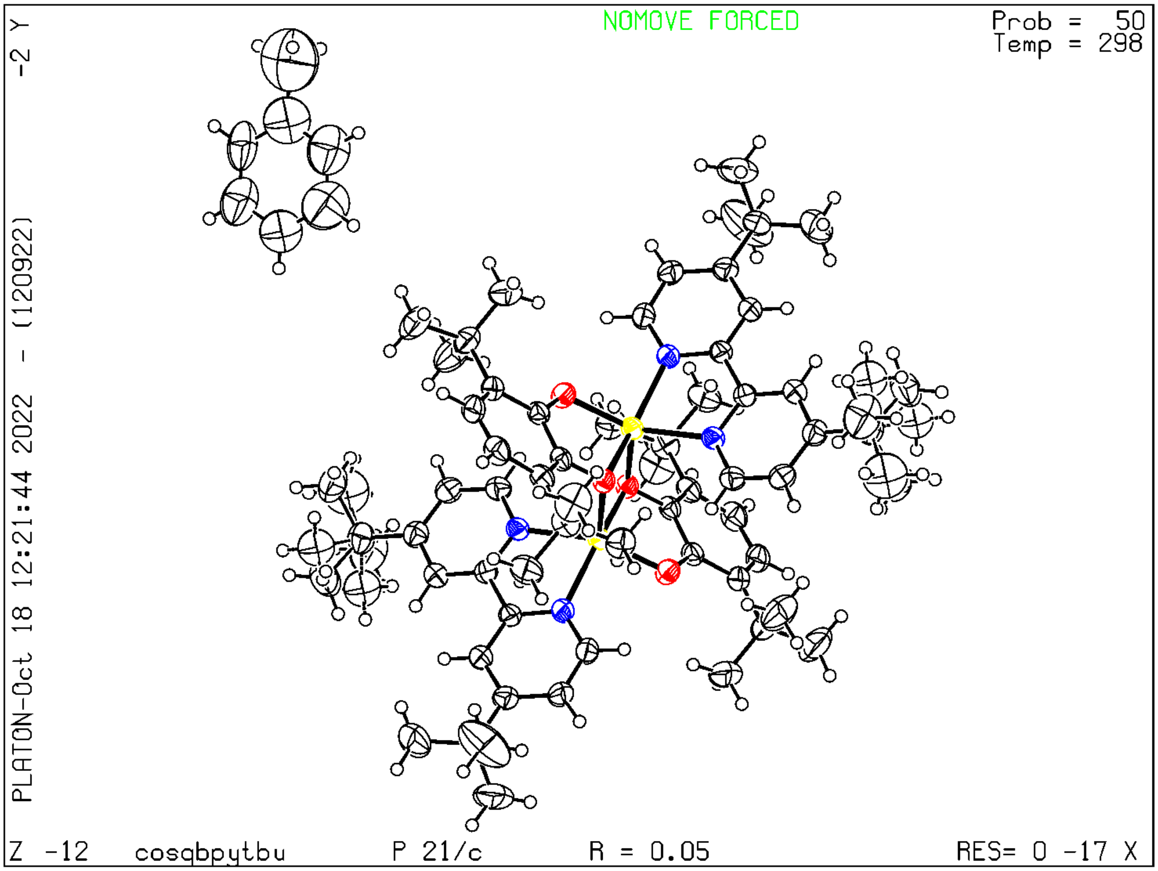

Supplement: Supplementary file 1 [file molecules-27-08175-s001.zip › complex 3.pdf]
